# Exfoliated Clay Decorated with Magnetic Iron Nanoparticles for Crystal Violet Adsorption: Modeling and Physicochemical Interpretation

**DOI:** 10.3390/nano10081454

**Published:** 2020-07-24

**Authors:** Mohamed Abou Elfetouh Barakat, Rajeev Kumar, Moaaz Korany Seliem, Ali Qurany Selim, Mohamed Mobarak, Ioannis Anastopoulos, Dimitrios Giannakoudakis, Mariusz Barczak, Adrián Bonilla-Petriciolet, Essam Abdelrahman Mohamed

**Affiliations:** 1 Department of Environmental Sciences, King Abdulaziz University, Jeddah 21589, Saudi Arabia; rsingh@kau.edu.sa; 2 Central Metallurgical R & D Institute, Helwan 11421, Cairo, Egypt; 3 Faculty of Earth Science, Beni-Suef University, Beni-Suef 62511, Egypt; Ali.qurany@esc.bsu.edu.eg (A.Q.S.); essam.abdelrahman@science.bsu.edu.eg (E.A.M.); 4 Physics Department, Faculty of Science, Beni-Suef University, Beni-Suef 62511, Egypt; Mohamed.mobarak@Lira.bsu.edu.eg; 5 Department of Chemistry, University of Cyprus, P.O. Box 20537, Nicosia Cy-1678, Cyprus; anastopoulos_ioannis@windowslive.com; 6 Institute of Physical Chemistry, Polish Academy of Sciences, Kasprzaka 44/52, 01-224 Warsaw, Poland; dgiannakoudakis@ichf.edu.pl; 7 Department of Theoretical Chemistry, Institute of Chemical Sciences, Faculty of Chemistry Maria Curie Skłodowska University in Lublin, 20-031 Lublin, Poland; mbarczak@umcs.pl; 8 Departamento de Ingeniería Química, Instituto Tecnológico de Aguascalientes, Aguascalientes 20256, Mexico; petriciolet@kbm.sdu.dk

**Keywords:** exfoliated clay, magnetic nanoparticles, dye adsorption, statistical modeling, desorption

## Abstract

Surfactant–modified exfoliated Fayum clay (CTAB–EC) obtained after chemical treatment with a CTAB/H_2_O_2_ solution was further decorated with magnetic Fe_3_O_4_ nanoparticles (MNP). The final nanocomposite (MNP/CTAB–EC) was characterized by XRD, SEM, FTIR, TEM and its adsorptive capability against a model cationic dye, crystal violet (CV), was evaluated. A comparison of the adsorption performance of the raw clay and its modified counterparts using H_2_O_2_, CTAB, CTAB/H_2_O_2_ or MNP indicated that the adsorption capacity of MNP/CTAB–EC was the highest for CV removal at pH 8.0. The pseudo‒second order for the kinetics and Freundlich model for adsorption equilibrium fitted well the CV removal experimental data at all tested temperatures (25, 40 and 55 °C). The enhancement of the Langmuir adsorption capacity from 447.1 to 499.4 mg g^−1^ with increasing the temperature from 25 to 55 °C revealed an endothermic nature of the removal process. The interactions between CV and MNP/CTAB–EC were interpreted using advanced statistical physics models (ASPM) in order to elucidate the adsorption mechanism. Multilayer model fitted the adsorption process and therefore, the steric and energetic factors that impacted the CV adsorption were also interpreted using this model. The aggregated number of CV molecules per MNP/CTAB–EC active site (n) was more than unity at all temperatures, representing thus a vertical adsorption orientation and a multi‒interactions mechanism. It was determined that the increase of CV uptake with temperature was mainly controlled by the increase of the number of active sites (*N*_M_). Calculated adsorption energies (Δ*E*) revealed that CV removal was an endothermic and a physisorption process (Δ*E* < 40 kJ mol ^−1^). MNP/CTAB–EC was magnetically separated, regenerated by NaOH, and reused without significant decrease in its adsorption efficiency, supporting a prosperity of its utilization as an effective adsorbent against hazardous dyes from wastewaters.

## 1. Introduction

Pollution of aquatic systems due to the industrial wastes associated with the creation and consumption of varied products such as paper, cotton, silk, wool, and leather, represents a global challenge [1,2]. The incessant release of toxic pollutants, including hazardous organic dyes, even at small concentrations, into water bodies is greatly harmful for human beings [3,4]. Crystal violet (CV), a common synthetic cationic dye, can be accumulated in the human body causing different diseases such as severe eye irritation, vomiting, tissue necrosis and even cancer [5]. Coagulation, precipitation, biological treatment and adsorption have been reported as alternative methods to reduce the concentrations of organic dyes in water [4]. Based on the removal efficacy, simplicity, and economic value, the water decontamination via the adsorption process is recommended [6]. The use of adsorbents based on available and low-cost materials such as clays and zeolites is reported to be a favorable method in water purification processes. In addition, those raw materials can be further modified through varied strategies to produce new composites with improved surface chemistry where several function groups (i.e., active adsorption sites) are available to interact with the water pollutants.

Recently, a solution of cetyltrimethylammonium bromide (CTAB) and hydrogen peroxide (H_2_O_2_) was used in the activation of the organic carbon–rich Fayum clay [7]. The increase of the positive charges on the modified clay surface resulted in improving its uptake efficiency, especially for anionic compounds such as hexavalent chromium and methyl orange [7]. Moreover, natural materials coated by magnetic iron oxides nanoparticles (MNP) were applied in the uptake of organic pollutants from water [4,8,9]. Modification of raw materials by MNP resulted in increasing their surface areas and adding new active adsorption sites, especially to interact with basic dyes [4,8]. In addition, the tested MNP adsorbents can be separated by a magnetic field to remove the attached organic contaminates and thus, it can be easily reused [4,8]. It should be remembered that CTAB itself is considered relatively toxic to aquatic organisms and may cause long-term adverse effects in the aquatic environment. Therefore, its sufficient binding to the sorbent should be ensured to avoid its release during the adsorption process. For instance, CTAB-assisted synthesis of ZIF-8 was applied as template to prepare ZIF-8–derived hollow carbon nanostructures as an adsorbent for antibiotics [10].

Herein, it is convenient to highlight that the description of the experimental data through the common classical models such as the Langmuir and Freundlich ones is a useful step for the identification of (I) the homogeneity or heterogeneity of the adsorbent active sites and (II) the number of adsorbate layers covering the adsorbent surface (i.e., one layer or multilayer). However, the parameters of these classical models are not appropriate for providing information about the steric and energetic parameters such as: the number of removed ions per active site (*n*), the density of active sites (*N_M_*), the adsorption capacity at saturation condition (*Q*_sat_) and the adsorption energy (-*ε*). Therefore, the advanced statistical physics models can be applied to determine these important adsorption parameters [2,9,11,12,13]. The interpretation of these parameters is crucial to understand the mechanism and the controlling factors of the adsorption process [4,12].

The main objectives of this study was (a) to synthesize a multifunctional composite via the decoration of CTAB‒exfoliated clay (CTAB‒EC) by magnetic nanoparticles (MNP), (b) to characterize this composite (MNP/CTAB‒EC) by different techniques including XRD, SEM, FTIR and TEM, (c) to describe the crystal violet (CV) adsorption results with kinetics and traditional isotherm models at different temperatures, and (d) to determine the physicochemical parameters associated with the adsorption of CV onto the MNP/CTAB‒EC composite via advanced statistical physics (ASPM) models.

## 2. Materials and Methods

### 2.1. Materials

A sample (i.e., 500 g) of representative natural Fayum clay (FC) was ground to pass through 100 mesh sieves and dried for 48 h at 105 °C. Cetyltrimethylammonium bromide (99% purity, Sigma-Aldrich, St. Louis, MO, USA), hydrogen peroxide (H_2_O_2_, 30%), ferric chloride (FeCl_3_·6H_2_O, Loba Chemie, Mumbai, India), iron sulfate (FeSO_4_·7H_2_O Loba Chemie, Mumbai, India), and ammonia hydroxide solution (NH_4_OH) were used as starting materials. Crystal violet (CV) with MF: C_25_N_3_H_30_Cl, MW: 408 was purchased from Fluka (Buchs, Switzerland) and used as received. Stock solutions (1 g L^−1^) of CV were prepared and the desired concentrations for adsorption experiments were obtained by diluting the stock solution with deionized water. The solution pH was attained by the addition of NaOH (0.01 mol/L) or HCl (0.01 mol/L).

### 2.2. Preparation of MNP/CTAB‒EC Adsorbent

Chemical activation of the FC sample with a mixture of CTAB and H_2_O_2_ was carried out as previously mentioned by Mobarak et al. [7]. This mixture was obtained by complete dissolving CTAB (1.275 g) in 50 mL of 30% H_2_O_2_ to obtain a CTAB/H_2_O_2_ solution. A portion of the FC sample (2.55 g) was mixed with CTAB/H_2_O_2_ solution and stirred at 50 °C for 2 h. The pH of the prepared mixture was adjusted to 12 using ammonia solution. At this solution pH, the adsorption of the positive CTAB onto the negative FC was significantly improved due to the presence of H_2_O_2_ that acts as an exfoliated agent [7]. The final product of CTAB and exfoliated Fayum clay (CTAB‒EC) were washed with ethanol/distilled water mixture five times and dried at 70 °C/24 h.

The chemical precipitation method [14] was used to prepare iron oxide nanoparticles (MNP) as follows: 1.05 g of FeSO_4_·7H_2_O and 2.1 g of FeCl_3_·6H_2_O were dissolved in 25 mL of distilled water with stirring for 30 min at room temperature (25 °C). Then, 10 mL of NH_4_OH was used as a precipitated agent (25%) and added with continuous stirring for 2 h. The preparation of magnetic nanoparticles (Fe_3_O_4_) was attained through the following reaction:

FeSO_4_·7H_2_O + 2FeCl_3_·6H_2_O + 8NH_4_OH → Fe_3_O_4_ +6NH_4_Cl + (NH4)_2_SO_4_ +17H_2_O (1)

The resulted magnetic nanoparticles were separated by magnet and mixed with CTAB‒EC at 50 °C for 2 h to produce the final product as given below:

Fe_3_O_4_ + CTAB‒EC→ MNP/CTAB‒EC (2)

The final composite (MNP/CTAB‒EC) was washed by distilled water and dried at 70 °C for 24 h to characterize and test for CV adsorption.

### 2.3. Sample Characterization

X–ray diffraction patterns of the raw and activated samples were recorded using an APD–3720 diffractometer (Philips) with Cu Kα radiation (40 kV, 40 mA) and wavelength (λ) = 1.54 Å. The scan angle was in the range of (5–80°) with a speed rate of 2°/min. The morphological features of the MNP/CTAB‒EC composite were observed via scanning electron microscopy (SEM, JSM-6700F, JEOL, Tokyo, Japan) and transmission electron microscope (JEM-2100F, JEOL, Tokyo, Japan). FTIR spectrum of the MNP/CTAB‒FC composite was recorded at room temperature in the region of 400–4000 cm^−1^ with a FTIR spectrometer (Vector 33 FTIR spectrometer, Bruker, Berlin, Germany). The values pH at the point of zero charge (pH_ZCP_) of the studied composite were determined as follows [15]: 20 mg of MNP/CTAB‒FC was added to 20 mL of KNO_3_ (0.1 mol L^−1^) at different values of pH in the range of 2.0–10.0 and shaken (100 rpm/24 h) at 25 °C. The difference between the initial pH (pH_i_) and final pH (pH_f_), was plotted against pH_i_. The pH_ZCP_ value is the point at which pH_f_–pH_i_ is equal to zero.

### 2.4. CV Adsorption Kinetics Using MNP/CTAB‒EC

Adsorption kinetics study of CV onto the studied adsorbent was run at three different temperatures, 25 °C, 40 °C, 55 °C, using a mixture of 25 mg of MNP/CTAB‒EC and 25 mL of CV with 250 mg L^−1^ of the basic dye concentration. CV and MNP/CTAB‒EC mixtures were shaken (200 rpm) by an orbital shaker (SHO‒2D, Berlin, Germany) at different times (i.e., 5, 30, 60, 120, 240, 360, and 480 min). CV concentrations in solutions obtained at each sampling time were measured by a double beam UV–vis spectrophotometer (Model UV 1601, Shimadzu, Tokyo, Japan). At each temperature, the removed amount ($q_{t}$) and the uptake percentage (R %) of CV were determined and λ_max_ = 580 nm from mass balance, using Equations (3) and (4):

(3) $$q_{t}\left(\mathrm{mg}\;g^{-1}\right)=\left(C_{0}–C_{t}\right)\frac{V}{m}$$

(4) $$R\;(\%)=\frac{100}{C_{0}}\left(C_{0}–C_{t}\right)$$

where $C_{0}$ and $C_{t}$ (mg L^−1^) are the initial and final CV concentrations after time (*t*), V is the CV solution volume (L) and m is the MNP/CTAB‒EC mass (g). In order to study the kinetics associated with CV uptake by MNP/CTAB‒EC adsorbent, the pseudo‒first order [16], the pseudo‒second‒order [17], and intra–particle diffusion [18] equations were used as listed in Table 1.

### 2.5. CV Adsorption Equilibrium

Adsorption isotherms were determined by adding 50 mg of the MNP/CTAB‒EC composite to 100 mL of CV with initial concentrations (200, 240, 280, 320, 360, and 400 mg L^−1^). The mixtures were shaken at 200 rpm for 2 h, which was identified as the equilibrium time, and the liquid phases were separated to determine the remaining CV concentration with UV–vis spectroscopy as indicated below. The CV adsorption capacity of MNP/CTAB‒EC was calculated at equilibrium (*q_e_*) as follows:

(5) $$q_{e}\left(\mathrm{mg}/g\right)=\left(C_{0}–C_{e}\right)\frac{\;V}{\;m}\;$$

where $C_{e}$ (mg L^−1^) is the equilibrium CV concentration in the solutions.

### 2.6. Traditional Modeling for CV Adsorption Onto MNP/CTAB‒EC

Langmuir [19], Freundlich [20], and Dubinin–Radushkevich [21] models were used to fit the adsorption isotherms of CV on the MNP/CTAB‒EC adsorbent, see Table 1. The determination coefficient ($R^{2}$) and the Chi–squared ($\chi^{2}$) values were used to find the best fit of tested traditional models [13]:

(6) $$R^{2}=1-\frac{\sum {\left(q_{e,\exp}\;-q_{e,\mathrm{cal}}\right)}^{2}}{\sum {\left(q_{e,\exp}-q_{e,\mathrm{mean}}\right)}^{2}}$$

(7) $$\chi^{2}=\sum \frac{{\left(q_{e,\exp}-q_{e,\mathrm{cal}}\right)}^{2}}{q_{e,\mathrm{cal}}}.$$

where $q_{e,\exp}\;\mathrm{and}\;q_{e,\mathrm{cal}}$ (mg·g^−1^) are the experimental and theoretical values of CV adsorption capacities, respectively.

### 2.7. Advanced Modeling for CV Adsorption Onto MNP/CTAB‒EC

To understand the CV adsorption mechanism at molecular level, a set of advanced statistical models (Table 2) was used to fit the CV adsorption results [4,10,13]. The best statistical physics model for CV adsorption onto MNP/CTAB‒EC composite was recognized depending on the calculated $R^{2}$ and the root mean square error (RMSE) calculated as follows [2,13]:

(8) $$\mathrm{RMSE}=\sqrt{\frac{\sum_{i=1}^{m}{\left(Q_{i\;\mathrm{cal}}-Q_{i\;\exp}\right)}^{2}}{m^{\prime }-p}}$$

where $m^{\prime }\;$denotes the experimental data and p is the number of adjustable parameters.

### 2.8. Regeneration of MNP/CTAB‒EC

CV desorption experiments were conducted at 25 °C using 100 mL NaOH (0.5 M) as a desorbing agent. MNP/CTAB‒EC loaded with CV were agitated on a rotatory shaker at 200 rpm for 2 h. After separation of MNP/CTAB‒EC, the concentration of CV in solution was determined following the experimental procedure already described. CV adsorption/desorption cycle was repeated five times. At the end of each adsorption cycle, the MNP/CTAB‒EC was washed five times by distilled water, dried at 65 °C for 8 h before the next desorption round.

## 3. Results and Discussions

### 3.1. Characterization of MNP/CTAB‒EC

**Figure 1:**
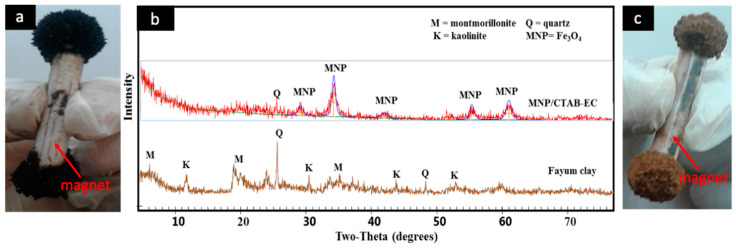
Photograph of magnetic nanoparticles (**a**), *X–ray diffraction* of the initial Fayum clay and the final composite MNP/CTAB-EC (**b**), and photograph of the final magnetic composite MNP/CTAB-EC (**c**).

The preparation of magnetic Fe_3_O_4_ nanoparticles (MNPs) was confirmed by the attraction of MNP towards a strong external magnet (Figure 1a). Figure 1b displays the XRD patterns of the investigated raw Fayum clay (FC) and the prepared MNP/CTAB‒EC composite. Montmorillonite and kaolinite (common clay minerals) as well as quartz (non‒clay mineral) were detected as main phases in FC [7]. Concerning the XRD pattern of MNP/CTAB‒EC, it can be observed the loss in the characteristic peaks of FC, especially the ones of the clay minerals. This could be related to the exfoliation towards an amorphous nature or/and to the deposition on the surface of the magnetic nanoparticles of Fe_3_O_4_ [22]. Furthermore, the diffraction peaks detected at 2θ = 30.58°, 35.76°, 43.62°, 57.25° and 63.05° can be associated to (220), (311), (400), (511) and (440) phases, respectively, which confirmed the retention of MNPs’ structure [9,22]. It should be mentioned that the XRD pattern of MNPs (data not presented) showed exactly the same above-mentioned reflections. As can be observed in Figure 1c, the composite material possesses also magnetic properties (Figure 1c).

**Figure 2:**
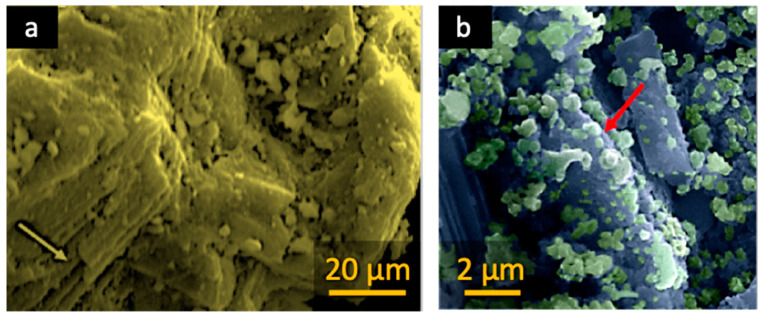

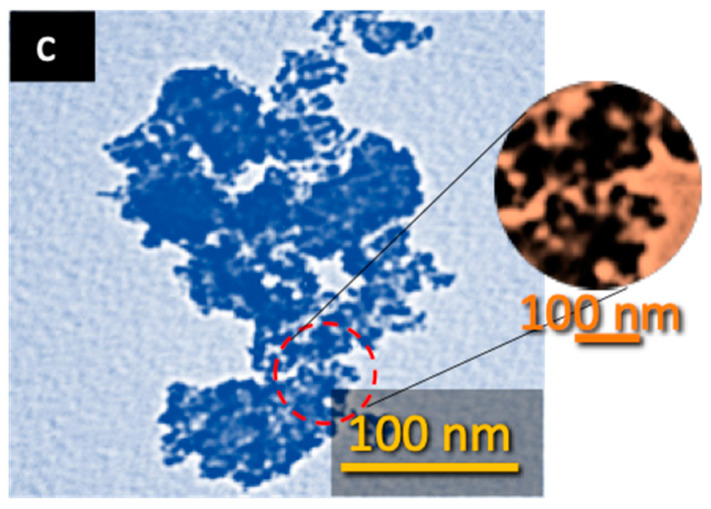
Scanning electron microscopy (SEM) images (**a**,**b**) and transmission electron microscopy (TEM) images (**c**) of MNP/CTAB–EC.

SEM analysis of MNP/CTAB‒EC revealed the existence of cages/voids in between the aggregated MNPs on the surface of the clay (Figure 2a,b). The presence of spherical particles contributes to increase the surface area for CV adsorption by MNP/CTAB‒EC and the availability of the potential adsorption sites [22]. The several observed cavities of the substrate can be associated with H_2_O_2_ activation/exfoliation. The presence of these voids, free or filled by MNP, can also favor the adsorptive capability of the final magnetic composite material.

The TEM image of MNP/CTAB‒EC (Figure 2c) shows clearly the existence of spherical-like nanoparticles, with diameter below 15 nm, anchored on the CTAB‒EC surface, thus reflecting a homogeneous dispersion.

**Figure 3:**
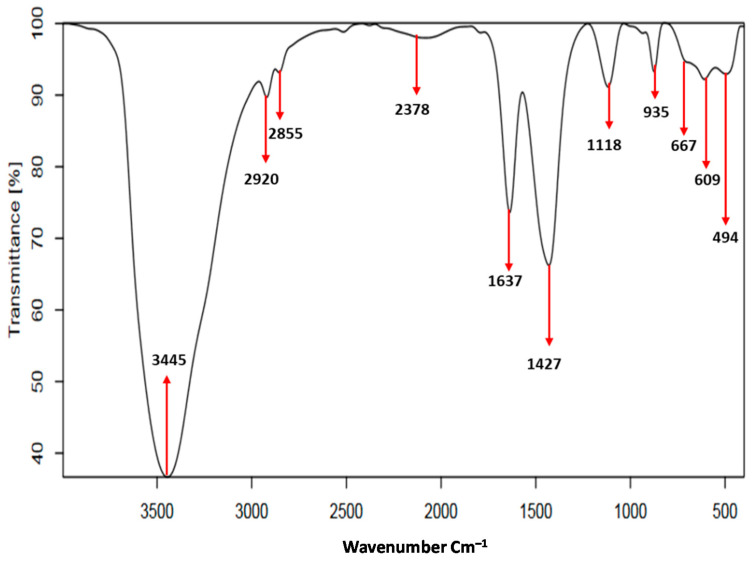
FTIR spectrum of MNP/CTAB-EC composite used in CV adsorption.

FTIR spectrum of MNP/CTAB‒EC (Figure 3) displays two strong bands at 3445 and 1637 cm^−1^ which are due to the bending and stretching vibrations of hydroxyl groups (‒OH) of physically attached H_2_O molecules [4,7]. In addition, the two bands detected at 2850 and 2920 cm^−1^ from the symmetric and asymmetric stretching vibrations of C–H group confirmed the addition of CTAB to the FC structure [15,23]. Furthermore, the characteristics bands for C–H group in MNP/CTAB‒EC were identified but their intensities were lower than those obtained to CTAB–EC synthesized in a previous study [7]. The decrease of these bands’ intensities could be associated with the covering by iron oxide nanoparticles [15]. The weak band observed at 2378 cm^−1^ was probably related to immersion of CO_2_ from the ambient atmosphere [24]. The bands located at 1427 and 667 cm^−1^ could be attributed to Fe–O group of MNP, which reflected the deposition of Fe_3_O_4_ on the CTAB‒EC surface [9,22]. The absorption bands at 1118 and 609 cm^−1^ were assigned to the stretching of Si–O–Si in silica [4]. The bands at 935 and 494 cm^−1^ were related to the bending vibrations of Al–OH and the condensed silica, respectively [22].

### 3.2. pH_ZCP_ of MNP/CTAB‒EC and pH Effect

**Figure 4:**
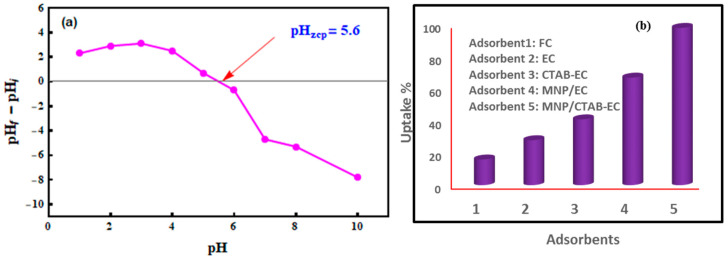
(**a**) pH at zero-point charge (pH _ZCP_) of MNP/CTAB–EC and (**b**) removal efficiency (%) of the FC, EC, CTAB-EC, MNP/EC and MNP/CTAB–EC for CV dye at pH 8.0.

The pH_ZCP_ was equal to 5.6 (Figure 4a) thus indicating that the MNP/CTAB‒EC surface could remove CV molecules more efficiently at pH > 6.0. To clarify that, the uptake percentage (%) of CV was tested at pH values of 5.0, 7.0, and 8.0 using 25 mg of MNP/CTAB‒EC, 50 mg L^−1^ of CV concentration, 2 h of contact time at 25 °C. The uptake results were 41.24, 95.56 and 96.80% at pH 5.0, 7.0 and 8.0, respectively. The electrostatic attraction between the deprotonated functional groups of MNP/CTAB‒EC composite and CV greatly enhanced the uptake percentage at pH 7.0 and 8.0. Therefore, all CV adsorption experiments were run at pH 8.0. Uptake of CV was also analyzed with raw Fayum clay (FC), H_2_O_2_‒activated clay (EC), surfactant‒modified EC (CTAB‒EC) and iron oxide nanoparticles coated EC (MNP/EC) under the same adsorption conditions (i.e., 25 mg of each adsorbent, 50 mg L^−1^ of CV concentration, 2 h of contact time at 25 °C, and pH 8.0). These adsorption studies confirmed the high uptake efficiency of MNP/CTAB‒EC in comparison with the other tested adsorbents (Figure 4b). The high efficiency of MNP/CTAB‒EC may be related to the high surface area and the great number of adsorption active sites (functional groups) of this adsorbent.

### 3.3. Interaction Time Effect on CV Uptake by MNP/CTAB‒EC

**Figure 5:**
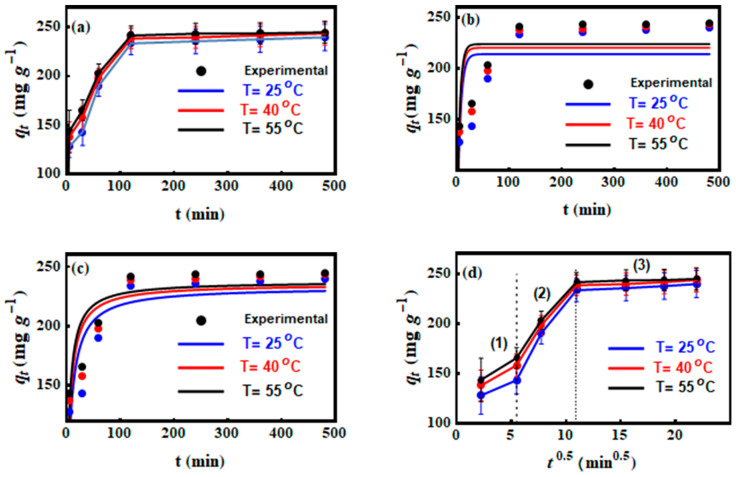
Kinetic studies of CV adsorption on MNP/CTAB–EC composite. (**a**) Effect of contact time, (**b**) Pseudo-first order model, (**c**) Pseudo-second order model and (**d**) intra-particle diffusion model at different temperatures (25, 40, and 55 °C).

CV adsorption onto MNP/CTAB‒EC was tested at different shaking times (from 5 to 480 min) and temperatures of 25, 40, and 55 °C. Results of these experiments are given in Figure 5a. The CV adsorption capacities ($q_{t}$) were 127.89, 137.89, and 143.48 mg g ^−1^ at 25, 40 and 55 °C, respectively, at the initial interaction times (i.e., up to the first 5 min) of uptake process. In the time interval of 0–5 min, the high adsorption of CV was due to the existence of abundant number of MNP/CTAB‒EC active sites (functional groups) available for CV removal [25]. From 5 to 120 min of dye‒adsorbent shaking time, the values of $q_{t\;}$were 233.52, 238.48, and 241.54 mg g ^−1^ at 25, 40 and 55 °C, respectively. The enhancement of $q_{t\;}$values could be associated to (I) the occupation of further active sites of MNP/CTAB‒EC surface by CV and (II) the diffusion of CV ions into the cavities and pores formed by activation process (i.e., intra‒particle diffusion CV transfer) [7,26,27]. From 120 to the end of interaction time (8 h), the $q_{t}\;$values of MNP/CTAB‒EC were nearly constant (Figure 5a) thus reflecting the equilibrium stage of CV adsorption [25].

### 3.4. CV Adsorption Kinetics

To determine the parameters of the pseudo-first-order and pseudo-second-order kinetic models, the non‒linear plots of $q_{t}$ versus time (Figure 5b,c) were used and the attained results are presented in Table 3. The coefficients of determination, *R^2^*, derived from fitting models have been provided to show differences in goodness of fitting. In the case of kinetics, pseudo-second-order model fitted the kinetics data in the best way (*R*^2^ is slightly higher). Moreover, the experimental and theoretical *q_e_* values obtained from the pseudo-second-order kinetic model were very similar (Table 3).

The plot of $q_{t}$ versus $t^{1/2}$ (Figure 5d) of the intra‒particle diffusion model was applied to discuss the diffusion mechanism of CV into the MNP/CTAB‒EC through the calculation of parameters$\;k_{p}$ and *C* as listed in Table 3. In the full-time range of 5–480 min, three common diffusion stages were exhibited (Figure 5d): (1) the external mass transfer, (2) pore‒diffusion and (3) equilibrium, respectively. Consequently, the CV adsorption onto MNP/CTAB‒EC was governed by more than one mechanism (i.e., surface diffusion and intra-particle diffusion) [4,12].

### 3.5. CV Adsorption Isotherms

Three common traditional equilibrium models (Langmuir, Freundlich, and D–R) and five advanced statistical physics models (monolayer with one energy site M1, monolayer with two energy sites M2, double layer with one energy site M3, double layer with two energy sites M4 and multilayer M5) were applied in fitting the experimental data of CV adsorption onto MNP/CTAB‒EC.

**Figure 6:**
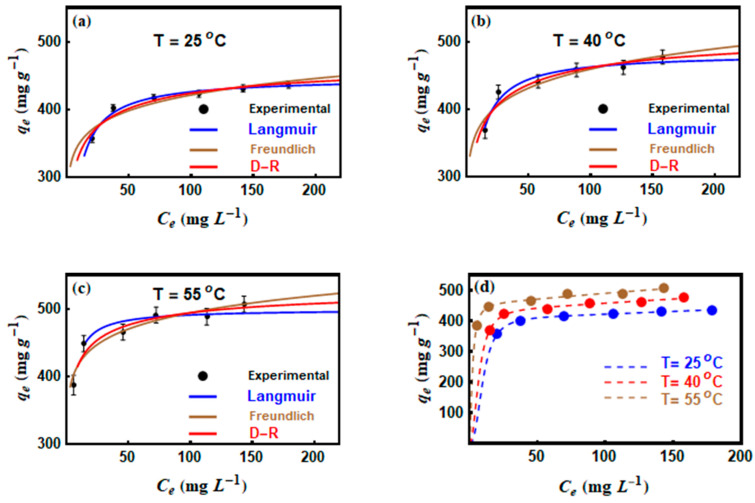
Results of (**a**–**c**) Langmuir, Freundlich and D-R isotherms and (**d**) statistical physics model for the CV adsorption on MNP/CTAB–EC composite at different temperatures (25, 40, and 55 °C).

The non–linear plot of $q_{e}$ against $C_{e}\;$(Figure 6) was used to determine the parameters of each applied model. According to the $R^{2}$ values, the three classical models fitted well the CV adsorption data ($R^{2}>0.99)\;$at all adsorption temperatures (25, 40, and 55 °C). Therefore, the Chi–squared ($\chi^{2}$) values were used to distinguish the best isotherm model. Freundlich equation showed the smallest $\chi^{2}\;$values at all temperatures, which reflected the applicability of this model in describing the CV adsorption results. The maximum Langmuir adsorption capacities ($q_{max}$) of CV were 447.08, 482.69, and 499.36 mg g ^−1^ at 25, 40, and 55 °C, respectively (Table 4). 

Thus, CV removal by this MNP/CTAB‒EC composite improved with increments on the adsorption temperature (i.e., endothermic adsorption) [4]. The increase of $K_{F}$ values from 289.91 to 354.82 mg g ^−1^ in tested temperature range (25–55 °C) confirming the endothermic nature of the adsorption process [28]. An endothermic nature of the adsorption processes of dyes on various sorbents (including clays) has been previously reported in literature [29,30,31]. A general explanation of the endothermic nature of dye adsorption is improving the mobility of dye molecules at higher temperatures, which leads to increased collision and binding of dye molecules to the adsorption sites [29]. Another reason may be that the increase in temperature leads to favorable intermolecular interactions between the dye and the adsorbent. Additionally, the values of 1/n were below unity (Table 4), which indicated a positive CV adsorption at low concentrations [10]. The E values ($E=\frac{1}{\sqrt{2\beta }}$) of the D–R model ranged from 5.93 to 7.85 kJ mol^−1^ by increasing the adsorption temperature from 25 to 55 °C and these results suggested that the interaction between CV and MNP/CTAB‒EC involved physical adsorption forces. Furthermore, the linear forms of the applied kinetics (the pseudo-first-order and pseudo-second-order) and the traditional equilibrium (Langmuir, Freundlich, and D–R) models were also involved, please see the supplementary data (Table S1 and Figures S1 and S2). The parameters of the kinetic and equilibrium adsorption models were listed in supplementary data, please see Table S2.

#### Advanced Statistical Physics Models (ASPM)

Based on the values of$\;R^{2}$ and RMSE (Table 5), the model M5 (i.e., multilayer adsorption model) was found to be the best one for representing the CV adsorption results. The steric and energetic parameters of this multilayer model were evaluated by a non-linear regression using the Wolfram Mathematica 10 program according to a 95% level of confidence.

According to M5, the CV adsorption was governed by different energies, which generally resulted in the formation of CV layers with a controlled number [4]. The first adsorption energy was attributed to the interaction between the first adsorbed CV layer (fixed number) and the MNP/CTAB‒EC active sites. On the other hand, the second adsorption energy was related to the dye‒dye interaction (i.e., CV‒CV interaction). As a result, the total number of the adsorbed CV layers is given by 1 + *N*_2_. Note that several operating scenarios can be considered to fit the multilayer model and to interpret the adsorption of CV on MNP/CTAB‒EC active sites as follows [4].

- Scenario 1: *n* and *N*_2_ are free adjustable parameters (i.e., multilayer).
- Scenario 2: *n* is a free adjustable parameter and *N*_2_ = zero (fixed) (i.e., a monolayer).
- Scenario 3: *n* is a free adjustable parameter and *N*_2_ = 1 (fixed) (i.e., double‒layer)
- Scenario 4: *n* is a free adjustable parameter and *N*_2_ = 2 (fixed) (i.e., triple‒layer)
- Scenario 5: *n* = unity (fixed) and *N*_2_ = zero (fixed) (i.e., Langmuir model)

These adsorption scenarios were analyzed, and the first scenario was selected to provide a theoretical interpretation of CV adsorption mechanism via steric and energy parameters.

### 3.6. Steric Parameters

#### 3.6.1. The n Parameter

**Figure 7:**
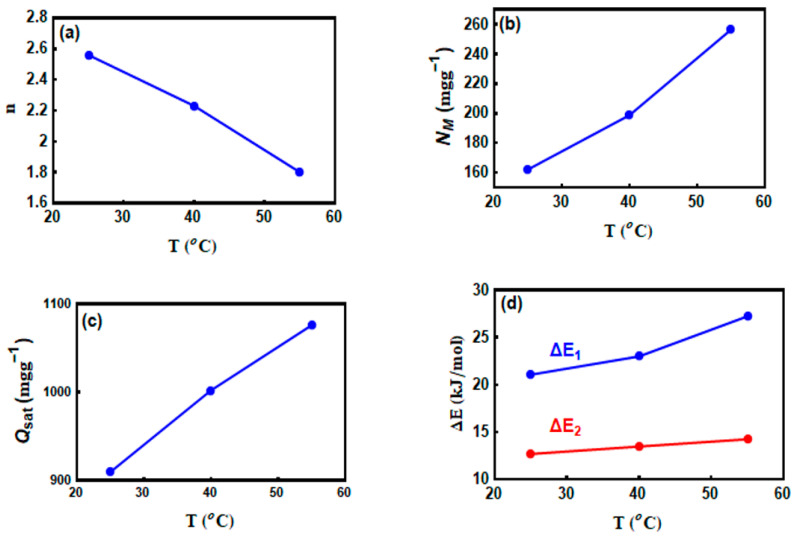
Evolution of statistical physics parameters (**a**) *n*, (**b**) *N_M_*, (**c**) *Q_sat_* and (**d**) ΔE as a function of temperature for the CV adsorption on MNP/CTAB–EC composite.

Overall, this parameter plays a major role in descriptive the geometry (horizontal or vertical) of the removed CV molecules on the MNP/CTAB‒EC surface. The horizontal or vertical adsorption orientation of the removed CV molecules are attributed to n with value more or lower than unity, respectively. Furthermore, the adsorption mechanism of the CV dye using MNP/CTAB‒EC adsorbent could be multi–docking (*n* < 1) or multi–molecular (*n* > 1) [2,10,11,32,33]. Consequently, various active sites of MNP/CTAB‒EC can adsorb one CV molecule when *n* < 1, while one adsorption site of this composite can adsorb numerous dye molecules if *n* > 1 [10]. Figure 7a shows the trend of parameter *n* as a function of solution temperature and Table 6 summarizes the values of this and remaining parameters of tested statistical physics model. The *n* parameter was 2.56, 2.23, and 1.8 at 25, 40, and 55 °C (i.e., all values were superior to 1). Thus, the vertical adsorption position and multi–molecular mechanism were involved during the interaction between CV molecules and MNP/CTAB‒EC active sites [10,32]. This result indicated that many CV molecules interacted with one MNP/CTAB‒EC active site (i.e., multi‒interactions mechanism) and, therefore, the adsorbed CV molecules were vertically oriented [11,32,33]. In general, three common cases can be used to describe the orientation of adsorbate onto adsorbent surfaces [32].

❖ Case 1 (*n* < 0.5): This scenario indicated that the CV molecule can interact with at least two adsorption sites of MNP/CTAB‒EC (i.e., a parallel adsorption orientation).
❖ Case 2 (0.5 < *n* < 1): This condition corresponded to parallel and non‒parallel orientations (mixed orientation with different percentages) for the adsorption of CV molecules.
❖ Case 3 (*n* > 1): This scenario indicated that CV molecules can interact with one active site of MNP/CTAB-EC (i.e., non-parallel adsorption orientation).

Since the values of *n* were within the range of 1.8 (dimer: *n* ~ 2) and 2.6 (trimer *n* ~ 3), see Table 6, it was conclude that the aggregated CV molecules interacted with one active adsorption site of MNP/CTAB‒EC surface, which implied a non‒parallel adsorption position.

#### 3.6.2. The N_M_ Parameter

Figure 7b shows the change of the MNP/CTAB‒EC active sites number (the *N_M_* parameter) with respect to the adsorption temperature. The values of *N_M_* were 162.24, 198.83, and 256.5 mg g^−1^ for CV adsorption at 25, 40 and 55 °C, respectively, see Table 6. The increment of the *N_M_* values could be attributed to the decrease of the *n* parameter. Ordinarily, the aggregation phenomenon associated with the adsorption process caused an increase of the *n* parameter and, consequently, a decrease in the number of the occupied MNP/CTAB‒EC active sites (i.e., a reduction of the *N_M_* parameter). Also, the increase of the *N_M_* values with temperature reflected the contribution of new active sites of this adsorbent (MNP/CTAB‒EC) in the CV adsorption process.

#### 3.6.3. Total Number of the Adsorbed CV Layers (N_t_ = 1+N_2_)

The determination of the total adsorbate layers is necessary to complement the understanding of the adsorption mechanism [32,33]. Calculated *N*_t_ values were 2.2, 2.3, and 2.33 for CV dye adsorption at 25, 40 and 55 °C, respectively (Table 6). The insignificant role of this parameter in controlling the adsorption process was recognized throughout the slight variation of *N*_t_ values at all tested temperatures. Consequently, the impact of the *N*_2_ parameter on the adsorption mechanism could be discarded.

#### 3.6.4. Adsorption Capacity of CV (Q_sat_ = n. N_M_. N_t_) at Saturation

The calculation of the values of *Q_sat_* is required to identify the removal efficacy of MNP/CTAB‒EC for the CV adsorption from aqueous solutions. Figure 7c displays the values of *Q_sat_* at all tested temperatures and a summary of these results are reported in Table 6. The *Q_sat_* values were 909.57, 1002.06, and 1075.76 mg g^−1^ for CV dye removal at 25, 40 and 55 °C, respectively. The interactions between CV molecules and MNP/CTAB‒EC was confirmed by the increment of *Q_sat_* values with temperature. The improvement of CV adsorption capacity with temperature could be related to the increment of the mobility of the dye molecules with temperature, which frequently allows the interaction of CV molecule with a great number of MNP/CTAB‒EC receptor sites [32]. As shown in Figure 7d, the *Q_sat_* values displayed the same trends identified for the steric parameters *N_M_* and *N*_t_ (i.e., the three parameters *Q_sat_*, *N_M_* and *N*_t_ increased with temperature), see Table 6. Based on the previous analysis, the effect of *N*_t_ on CV uptake can be neglected and, thus, the adsorption capacity of this dye was mainly associated with the parameter *N_M_*. Previous studies have reported maximum CV adsorption capacities of 47.27, 64.93, 44.88, 4.72 and 248.62 mg g^−1^ for kaolin, treated ginger waste, modified rice husk, clay/polymer composite, and black limestone, respectively [13]. This comparison indicated that MNP/CTAB‒EC can be recommended as an effective adsorbent for the treatment of textile effluents contaminated by CV dye.

### 3.7. CV Adsorption Energy (ΔE)

The values of adsorption energies play a significant role to interpret the interaction between CV molecules and MNP/CTAB‒EC active sites [9]. The calculation of these adsorption energies was given by:

(9) $$C_{1}=C_{s}e^{-\frac{\Delta E_{1}}{RT}}$$

(10) $$C_{2}=C_{s}e^{-\frac{\Delta E_{2}}{RT}}$$

where $c_{1}$and $c_{2}$ are the concentrations at half-saturation and $c_{s}\;$indicates the CV solubility.

Figure 7d and Table 6 show the calculated adsorption energies (Δ*E*) of CV as a function of the solution temperature. The calculated Δ*E* values were below 40 kJ mol^−1^ at all temperatures, which reflected a physical adsorption process [32]. The values of Δ*E*_1_ were higher than those of Δ*E*_2_ at 25, 40 and 55 °C as expected (see Table 6). The high values of Δ*E*_1_ were attributed to the strong interactions between MNP/CTAB‒EC active sites and CV molecules, while Δ*E*_2_ implied the CV‒CV interactions. Energetically, the observed Δ*E* trend was similar to that of *Q_sat_* (i.e., Δ*E* and *Q_sat_* increased with temperature increments), see Table 6. Consequently, the mechanism of CV adsorption onto MNP/CTAB‒EC composite was governed by steric (*N_M_*) and energetic (Δ*E*) parameters.

**Figure 8:**
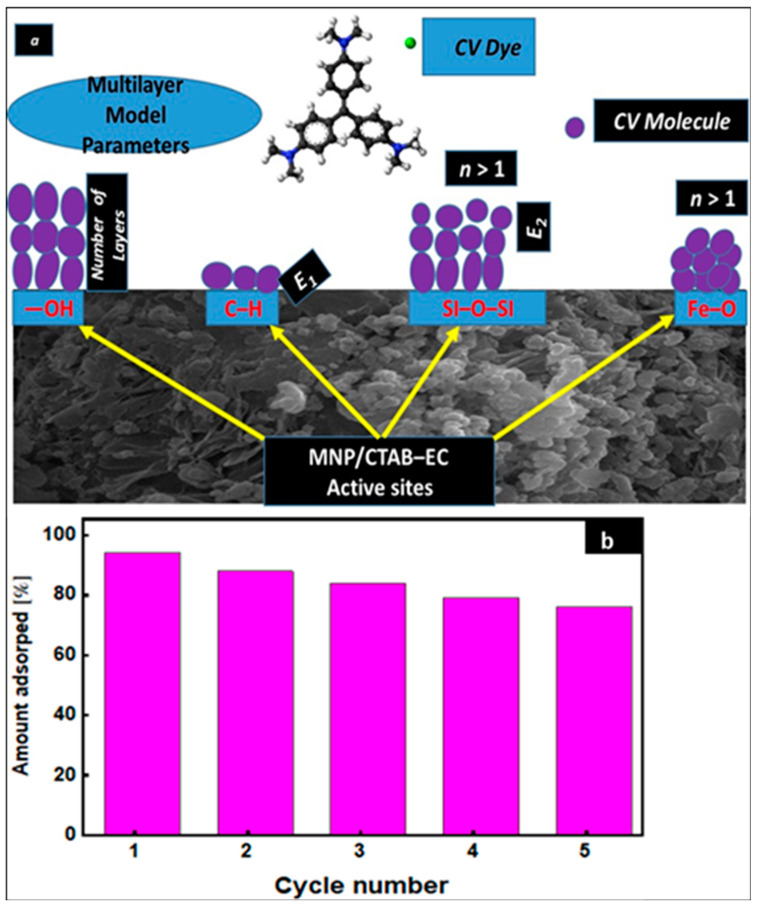
(**a**) Proposal of CV adsorption mechanism based on the steric and energetic parameters of the multilayer model and (**b**) relative uptake percentages after MNP/CTAB–EC regeneration.

Figure 8a displays a proposal of the CV adsorption mechanism onto MNP/CTAB‒EC active sites based on the steric and energetic parameters of the multilayer adsorption model.

### 3.8. Reusability Study

The possibility of multiple applications of an adsorbent is considered as one of the most important issues in adsorption-based industrial processes [34,35,36,37,38,39]. In the current study, five repetition cycles of adsorption/desorption were conducted to evaluate the regeneration of MNP/CTAB‒EC adsorbent. CV dye removal with MNP/CTAB‒EC was 92.88, 88.76, 83.65, 79.18, and 76.54% after cycles 1, 2, 3, 4, and 5, respectively (cf. Figure 8b). This regeneration study revealed that the MNP/CTAB‒EC adsorbent can be reused several times in the CV dye adsorption thus providing an additional economic benefit. A slight decrease in adsorption capacities is observed after each adsorption-desorption cycle, most probably due to the gradual degradation of the sorbent [40], particularly its effective adsorption sites [41] during adsorption and regeneration with NaOH. Another reasons may be related to the irreversible blockage of some adsorption sites and then, CV is not removed during regeneration.

### 3.9. Comparison with Other Adsorbents

Table 7 shows a comparison of the adsorption capacities of various reported sorbents; as can be seen, the maximum sorption capacity of MNP/CTAB‒EC adsorbent is comparable to other adsorbents and was found to be higher than for many sorbents reported in the literature. Therefore, the synthetic MNP/CTAB‒EC is recommended to be a promising adsorbent material for CV uptake from contaminated solutions.

Finally, the attained results through the current study confirmed the high uptake capacities of nanoparticles and carbon-based adsorbents against different water contaminates including organic dyes [42,43].

## 4. Conclusions

MNP/CTAB‒EC composite was synthesized and applied for the CV adsorption from aqueous solutions. The pseudo‒second‒order and Freundlich models fitted the CV adsorption on MNP/CTAB‒EC at 25, 40, and 55 °C, while a multilayer statistical physics model was the best one to correlate and interpret the experimental CV adsorption data. Steric and energetic parameters associated with a multilayer adsorption model indicated that CV molecules were adsorbed in a vertical position on the adsorbent surface and governed by multi–interactions mechanisms. Sterically, the parameter *N_M_* displayed the most important role in managing the CV adsorption process. The values of adsorption energies indicated that the CV uptake by MNP/CTAB‒EC adsorbent is endothermic process mainly governed by physical interactions. This new composite can be easily reused several times without a significant loss of its adsorption. This study clearly shows that the obtained MNP/CTAB‒EC material can be applied as for efficient removal of crystal violet (as well as the other dyes) from contaminated waters and wastewaters.

## Figures and Tables

**Table 1:** Kinetic and isotherm models used to fit the adsorption of CV on the MNP/CTAB-EC composite.

Kinetic Model	Equation	Parameters	Ref.
Pseudo- first order	$q_{t}=q_{e}\left(1-e^{-k_{1}t}\right)$	$q_{t}$ (mg g^−1^) is the removed amount of CV at time t.	[16]
		$q_{e\;}$(mg g^−1^) is the equilibrium adsorption capacity.	
		$k_{1\;}$(g mg^−1^ min^−1^) is the rate constant of the first-order adsorption.	
Pseudo- second order	$q_{t}=\frac{q_{e}^{2}\;k_{2}\;t}{q_{e}\;k_{2}\;t+1}$	$k_{2\;}$(g mg^−1^ min^−1^) is the rate constant of the second-order adsorption	[17]
Intra-particle diffusion	$q_{t}=k_{p}\;t^{1/2}+C$	$k_{p}\;$(mg g^−1^ min^0.5^) is the intraparticle diffusion rate constant.	[18]
		*C* (mg g^−1^) is the intercept of the line.	
Isotherm Model			
		$C_{e}$ (mg L^−1^) is the equilibrium concentration of the CV in the solution	[19]
Langmuir	$q_{e}=\frac{q_{max\;K_{L}C_{e}}}{\left(1+K_{L}C_{e}\;\right)}$	$q_{e}\;$(mg g^−1^) is the removed amount of CV at equilibrium.	
		$q_{max}$ (mg g^−1^) is the maximum adsorption capacity	
		$K_{L}$ (L mg^−1^): is the Langmuir constant	
		$K_{F}$ (mg g^−1^) is the CV adsorption capacity.	[20]
Freundlich	$q_{e}=K_{F}$ $C_{e}{}^{1/n}$	*n* is the heterogeneity factor.	
		β (mol^2^/kJ^2^) is the D-R constant	[21]
Dubinin–Radushkevich	$q_{e}=q_{m\;}e^{-\beta \varepsilon^{2}}$	ε (kJ^2^/mol^2^) is the Polanyil potential, equal to $RT\ln\left(1+\frac{1}{C_{e}}\right)$	
		R is the universal gas constant (8.31 J (mol K)^−1^).	
		*T* (K) is the absolute temperature.	
		$q_{m}$ (mg g^−1^) is the theoretical adsorption capacity.	

**Table 2:** Advanced statistical physics models to analyze the adsorption of CV on MNP/CTAB-EC composite.

Advanced Statistical Physics Modes			Ref.
(M 1) Monolayer with one energy	$Q=nN_{o}=\frac{nN_{M}}{1+{\left(\frac{c_{1/2}}{c}\right)}^{n}}=\frac{Q_{o}}{1+{\left(\frac{c_{1/2}}{c}\right)}^{n}}$	*Q* (mg g^−1^) is the adsorbed quantity, *n* is the ions number per site, *N_M_* (mg g^−1^) is the receptor sites density, *Q*_o_ (mg g^−1^) is the adsorbed quantity at saturation. $c_{1/2}$ (mg L^−1^) is the concentration at half–saturation.	[11]
(M 2) Monolayer with two energies	$Q=\frac{n_{1}N_{1M}}{1+{\left(c_{1}/c\right)}^{n_{1}}}+\;\frac{n_{2}N_{2M}}{1+{\left(c_{2}/c\right)}^{n_{2}}}$	*c*_1_ and *c*_2_ (mg L^−1^) are the concentrations at half saturation for the first and the second active sites, respectively. *n*_1_ and *n*_2_ (–) are the ions number per site for the first and the second receptor sites, respectively.	[13]
(M 3) Double layer with one energy	$Q=Q_{o}\frac{{\left(\frac{c}{c_{1/2}}\right)}^{n}+2{\left(\frac{c}{c_{1/2}}\right)}^{2n}}{1+{\left(\frac{c}{c_{1/2}}\right)}^{n}+{\left(\frac{c}{c_{1/2}}\right)}^{2n}}$		[13]
(M 4) Double layer with two energies	$Q=Q_{o}\frac{{\left(\frac{c}{c_{1}}\right)}^{n}+2{\left(\frac{c}{c_{2}}\right)}^{2n}}{1+{\left(\frac{c}{c_{1}}\right)}^{n}+{\left(\frac{c}{c_{2}}\right)}^{2n}}$		[13]
(M 5) multilayer	$Q=n\;N_{M}\frac{F_{1}\left(c\right)+F_{2}\left(c\right)+F_{3}\left(c\right)+F_{4}\left(c\right)}{G\left(c\right)}$ where $F_{1}\left(c\right)=-\frac{2{\left(\frac{c}{c_{1}}\right)}^{2n}}{1-{\left(\frac{c}{c_{1}}\right)}^{n}}+\frac{{\left(\frac{c}{c_{1}}\right)}^{n}\left(1-{\left(\frac{c}{c_{1}}\right)}^{2n}\right)}{{\left(1-{\left(\frac{c}{c_{1}}\right)}^{n}\right)}^{2}},$ $F_{2}\left(c\right)=\frac{2{\left(\frac{c}{c_{1}}\right)}^{n}{\left(\frac{c}{c_{2}}\right)}^{n}\left(1-{\left(\frac{c}{c_{2}}\right)}^{n\;N_{2}}\right)}{1-{\left(\frac{c}{c_{2}}\right)}^{n}},$ $F_{3}\left(c\right)=-N_{2}\frac{{\left(\frac{c}{c_{1}}\right)}^{n}{\left(\frac{c}{c_{2}}\right)}^{n}{\left(\frac{c}{c_{2}}\right)}^{n\;N_{2}}}{1-{\left(\frac{c}{c_{2}}\right)}^{n}},$ $F_{4}\left(c\right)=\frac{{\left(\frac{c}{c_{1}}\right)}^{n}{\left(\frac{c}{c_{2}}\right)}^{2n}\left(1-{\left(\frac{c}{c_{2}}\right)}^{n\;N_{2}}\right)}{{\left(1-{\left(\frac{c}{c_{2}}\right)}^{n}\right)}^{2}},$ $G\left(c\right)=\frac{\left(1-{\left(\frac{c}{c_{1}}\right)}^{2n}\right)}{1-{\left(\frac{c}{c_{1}}\right)}^{n}}+\frac{{\left(\frac{c}{c_{1}}\right)}^{n}{\left(\frac{c}{c_{2}}\right)}^{n}\left(1-{\left(\frac{c}{c_{2}}\right)}^{n\;N_{2}}\right)}{{\left(1-{\left(\frac{c}{c_{2}}\right)}^{n}\right)}^{2}},$		[13]

**Table 3:** Parameters of kinetic models for the adsorption of CV on MNP/CTAB-EC composite.

Kinetic Model	*T =* 25 °C	*T =* 40 °C	*T =* 55 °C
Pseudo-first-order			
$q_{e(\exp)}\;$(${\mathrm{mg}\;g}^{-1}$)	239.72	243.8	244.9
$q_{e(\mathrm{cal})}\;$(${\mathrm{mg}\;g}^{-1}$)	214.27	220.52	224.07
$k_{1}\;$ (g (mg min)^−1^)	0.17	0.19	0.20
$R^{2}$	0.9747	0.9811	0.9837
Pseudo-second-order			
$q_{e(\mathrm{cal})}\;$(${\mathrm{mg}\;g}^{-1}$)	232.92	235.48	237.59
$k_{2}\;$ (g (mg min)^−1^)	0.00062	0.00081	0.00091
$R^{2}$	0.9871	0.9901	0.9915
Intra-particle diffusion			
$k_{p}$ (mg(g min^0.5^)^−1^)	5.899	5.442	5.18
*C* (${\mathrm{mg}\;g}^{-1}$)	131.36	143.92	150.97
$R^{2}$	0.7901	0.7899	0.7782

**Table 4:** Parameters of isotherms models for the adsorption of CV on MNP/CTAB-EC composite.

Isotherm Model	*T =* 25 °C	*T =* 40 °C	*T =* 55 °C
Langmuir			
$q_{max}$(${\mathrm{mg}\;g}^{-1}$)	447.08	482.69	499.36
$k_{L}\;$ (${L\;\mathrm{mg}}^{-1})$	0.203	0.23	0.594
$R^{2}$	0.9999	0.9997	0.9996
$\chi^{2}$	0.21	0.77	0.97
Freundlich			
$k_{F}\;\left({\mathrm{mg}\;g}^{-1}\right)$	289.912	301.84	354.82
1/n	0.081	0.091	0.072
$R^{2}$	0.9996	0.9993	0.9994
$\chi^{2}$	1.135	1.95	1.8
D−R			
$q_{m}\left({\mathrm{mg}\;g}^{-1}\right)$	463.8	506.22	525.45
$E\left({\mathrm{kJ}\;\mathrm{mol}}^{-1}\right)$	5.93	6.38	7.85
$R^{2}$	0.9998	0.9995	0.9996
$\chi^{2}$	0.617	1.305	1.146

**Table 5:** Values of determination coefficients *R*^2^ and RMSE for the tested isotherms models for the adsorption of CV on MNP/CTAB–EC composite.

T (°C)	25		40		55	
	*R* ^2^		*R* ^2^		*R* ^2^	
	RMSE		RMSE		RMSE	
M 1	0.99994	0.14	0.9997	0.77	0.9997	0.93
M 2	0.99997	0.07	0.9998	0.43	0.9998	0.57
M 3	0.99995	0.13	0.9997	0.66	0.9997	0.81
M 4	0.99995	0.12	0.9997	0.67	0.9997	0.82
M 5	0.99999	0.003	0.9999	0.2	0.9999	0.37

**Table 6:** Steric and energetic parameters of the multilayer layer model for the adsorption of CV on MNP/CTAB-EC composite.

T	n	$N_{M}$	$1+N_{2}$	$\Delta E_{1}$	$\Delta E_{2}$	$Q_{sat}$
(°C)	(–)	(mg g^−1^)	(mg g^−1^)	(kJ mol^−1^)	(kJ mol^−1^)	(mg g^−1^)
25	2.56	162.24	2.19	21.06	12.69	909.57
40	2.23	198.83	2.26	22.99	13.47	1002.06
55	1.8	256.5	2.33	27.22	14.24	1075.76

**Table 7:** Comparison of the maximum adsorption capacities ($q_{max}$) of various reported sorbents for CV.

Sorbent	*q_max_* (mg g^−1^)	Ref.
Alginate/Pectin nanocomposite	619	[44]
BaCO_3_/g-C_3_N_4_	1240	[45]
*Ocotea puberula* bark powder	444	[46]
Black limestone	340	[47]
Carbon nanotubes modified with deep eutectic solvent	394	[48]
Magnetic chitosan nanocomposite	77	[49]
Polymer-based hydrogel	453	[50]
ZSM-5 zeolite		[51]
Chitin-templated ZSM-5 zeolite	1217	[51]
Mango stone biocomposite	353	[52]
MCM-41 silica	237	[53]
Palygorskite clay	53	[54]
SBA-15 nanoparticles	588	[41]
MNP/CTAB‒EC	448	This work

## Supplementary Materials

The following are available online at https://www.mdpi.com/2079-4991/10/8/1454/s1, Figure S1: Linear forms of the Pseudo-first order model (**a**) and the Pseudo-second order model (**b**) for CV uptake by MNP/CTAB-EC composite at different temperatures, Figure S2: Linear Langmuir, Freundlich, and D–R isotherms models for CV uptake by MNP/CTAB-EC composite at different temperatures, Table S1: Kinetic and isotherm linear models for CV uptake by MNP/CTAB-EC composite., Table S2: Parameters of linear of kinetic and isotherm models for the adsorption of CV onto MNP/CTAB-EC composite.
