# Supplementary material for: Exfoliated Clay Decorated with Magnetic Iron Nanoparticles for Crystal Violet Adsorption: Modeling and Physicochemical Interpretation
Source: Nanomaterials (Basel). 2020 Jul 24;10(8):1454. doi: 10.3390/nano10081454 (PMC7466639; doi:10.3390/nano10081454)
Supplement: Supplementary file 1 [file nanomaterials-10-01454-s001.pdf]

nanomaterials

MDPI

Supplementary Materials

Exfoliated Clay Decorated with Magnetic Iron Nanoparticles for Crystal Violet Adsorption: Modeling and Physicochemical Interpretation

Mohamed Abou Elfetouh Barakat [1,2,*], Rajeev Kumar [1], Moaaz Korany Seliem [3,*], Ali Qurany Selim [3], Mohamed Mobarak [4], Ioannis Anastopoulos [5], Dimitrios Giannakoudakis [6], Mariusz Barczak [7], Adrián Bonilla-Petriciolet [8] and Essam Abdelrahman Mohamed [3]

[1] Department of Environmental Sciences, King Abdulaziz University, Jeddah 21589, Saudi Arabia; rsingh@kau.edu.sa
[2] Central Metallurgical R & D Institute, Helwan 11421, Cairo, Egypt
[3] Faculty of Earth Science, Beni-Suef University, Beni-Suef 62511 Egypt; Ali.qurany@esc.bsu.edu.eg (A.Q.S.); essam.abdelrahman@science.bsu.edu.eg (E.A.M.)
[4] Physics Department, Faculty of Science, Beni-Suef University, Beni-Suef 62511 Egypt; Mohamed.mobarak@Lira.bsu.edu.eg
[5] Department of Chemistry, University of Cyprus, P.O. Box 20537, Nicosia Cy-1678, Cyprus; anastopoulos_ioannis@windowslive.com
[6] Institute of Physical Chemistry, Polish Academy of Sciences, Kasprzaka 44/52, 01-224 Warsaw, Poland; dgiannakoudakis@ichf.edu.pl
[7] Department of Theoretical Chemistry, Institute of Chemical Sciences, Faculty of Chemistry Maria Curie Skłodowska University in Lublin, 20-031 Lublin, Poland; mbarczak@umcs.pl
[8] Departamento de Ingeniería Química, Instituto Tecnológico de Aguascalientes, Aguascalientes 20256, Mexico; petriciolet@kbm.sdu.dk
* Correspondence: mabarakat@gmail.com (mababdullah1@kau.edu.sa) (M.A.E.B.); moaaz.korany@science.bsu.edu.eg (M.K.S.)

Table S1. Kinetic and isotherm linear models for CV uptake by MNP/CTAB-EC composite.

Kinetic Model	Linear	Parameters	Refs.
Pseudo first-order	$ln(q_e - q_t) = ln\, q_e - k_1 t$	q_t (mg g^{-1}) is the removed amount of CV at time t. q_e is the equilibrium adsorption uptake (mg g^{-1}). k_1 is the rate constant of the first-order adsorption (min)$^{-1}$.	[16]
Pseudo Second-order	$\frac{t}{q_t} = \frac{1}{k_2\, q_e^2} + \frac{t}{q_e}$	k_2 is the rate constant of the second-order adsorption (g(mg min)$^{-1}$).	[17]
Isotherm Model			
Langmuir	$\frac{C_e}{q_e} = \frac{1}{q_{\max}\, b} + \frac{C_e}{q_{\max}}$	C_e (mg L^{-1}): equilibrium concentration of the CV in the solution q_e (mg g^{-1}): removed amount of CV at equilibrium. $q_{\max}$ (mg g^{-1}): maximum adsorption capacity K_L (L mg^{-1}): Langmuir constant	[19]
Freundlich	$log\, q_e = log\, K_F + \frac{1}{n} log\, C_e$	K_F (mg g^{-1} (mg L^{-1})$^{-1/n}$) CV adsorption capacity. n: heterogeneity factor.	[20]
D–R	$ln\, q_e = ln\, q_m - \beta\varepsilon^2$	β(mol^2/kJ^2): D–R constant ε (kJ^2/mol^2): Polanyil potential, equal to $RT\, ln\left(1 + \frac{1}{C_e}\right)$. R: universal gas constant (8.31 J/mol K). T (K): absolute temperature. q_m (mg g^{-1}): theoretical adsorption capacity.	[21]

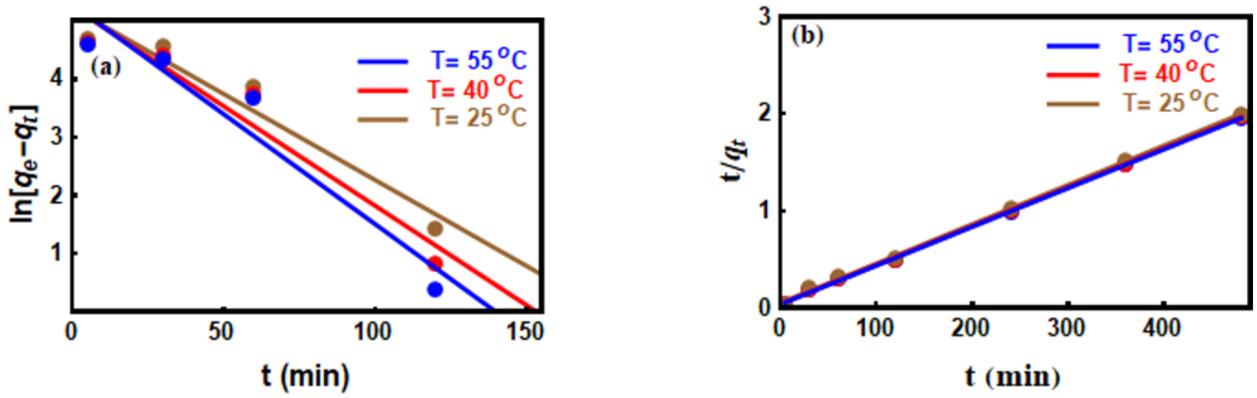

Figure S1. Linear forms of the Pseudo-first order model (**a**) and the Pseudo-second order model (**b**) for CV uptake by MNP/CTAB-EC composite at different temperatures.

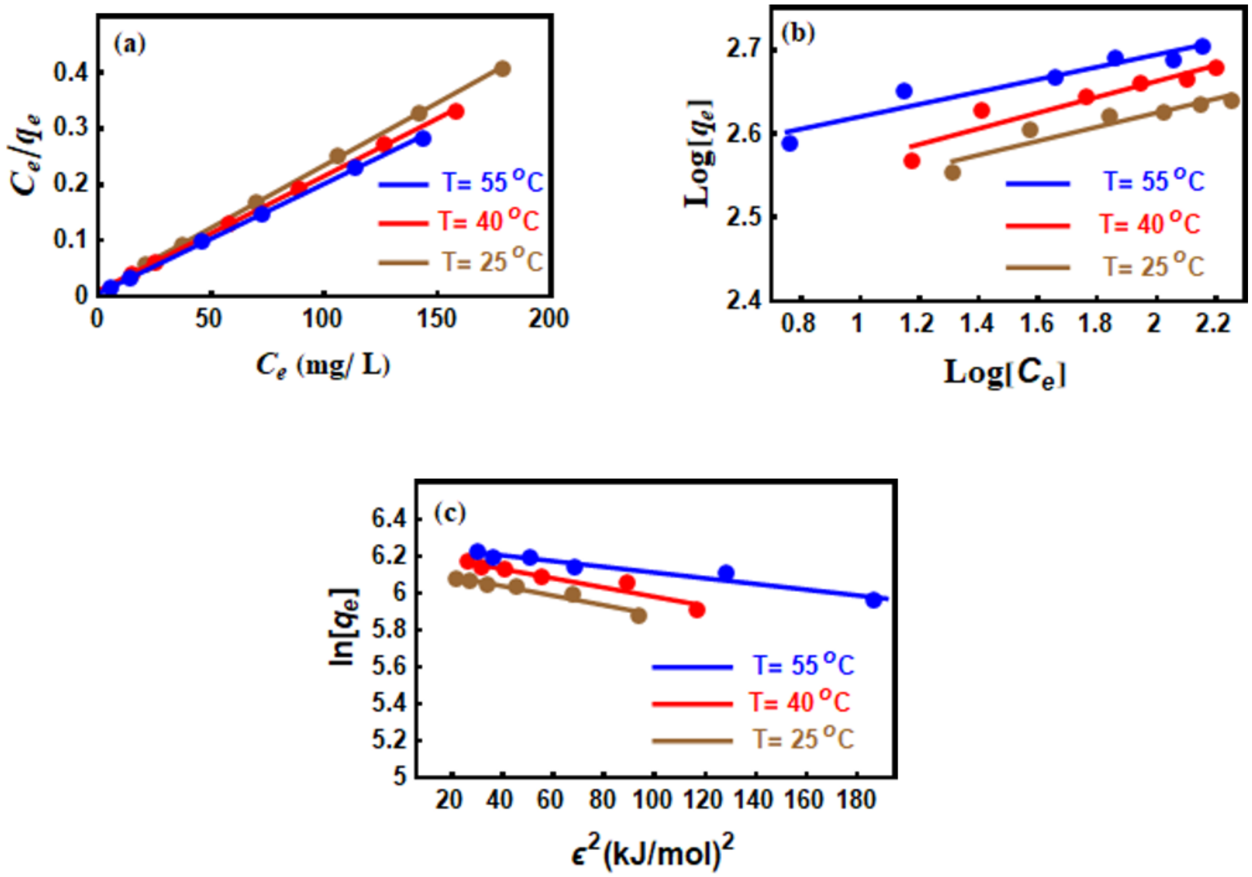

Figure S2. Linear Langmuir, Freundlich, and D–R isotherms models for CV uptake by MNP/CTAB-EC composite at different temperatures.

Table S2. Parameters of linear of kinetic and isotherm models for the adsorption of CV onto MNP/CTAB-EC composite.

**Kinetic Model**	**T = 25 °C**	**T = 40 °C**	**T = 55 °C**
Pseudo-first-order			
$q_{e(exp)}$ (mg g^{-1})	239.72	243.8	244.9
$q_{e(cal)}$ (mg g^{-1})	185.82	189.94	198.28
k_1 (min^{-1})	0.03	0.034	0.038
R^2	0.9401	0.9315	0.9247
Pseudo-second-order			
$q_{e(cal)}$ (mg g^{-1})	246.37	249.22	250.1
k_2 (g (mg min)$^{-1}$)	0.00032	0.00038	0.00044
R^2	0.9989	0.9993	0.9994

Isotherm Model			
Langmuir			
q_{max} (mg g^{-1})	447.41	486.76	509.11
k_L (L mg^{-1})	0.2	0.21	0.37
R^2	0.9998	0.9992	0.9996
Freundlich			
k_F (mg g^{-1})	286.5	297.98	351.96
$1/n$	0.08	0.09	0.07
R^2	0.8925	0.8881	0.913
D−R			
q_{m}(mg g^{-1})	465.01	507.77	526.36
E(kJ mol^{-1})	13.89	14.16	17.99
R^2	0.9414	0.9252	0.9458
